# Reply to Gong et al.: Antibiotic tolerance coupled with limited benefit from drainage: Clinical challenges in *Klebsiella pneumoniae* liver abscess

**DOI:** 10.1073/pnas.2615420123

**Published:** 2026-08-03

**Authors:** Sarah E. Rowe

**Affiliations:** ^a^Department of Microbiology and Immunology, University of North Carolina, Chapel Hill, NC 27599

We thank Gong and colleagues for their thoughtful Letter (1) and for their interest in our recent study of treatment failure in *Klebsiella pneumoniae* liver abscesses (KLAs) (2). We are encouraged that their clinical observations align with our central finding that antibiotic tolerance, rather than insufficient drug exposure, is a major contributor to therapeutic failure in these infections.

Gong et al. present clinical data demonstrating that antibiotic concentrations within abscess material can exceed pharmacodynamic targets despite limited response to antimicrobial therapy (1). These findings are highly consistent with our experimental results, in which we directly quantified antibiotic concentrations in infected liver tissue and used imaging mass spectrometry to visualize drug distribution within abscesses. In our study, antibiotics were detected at bactericidal concentrations within abscess regions, yet viable bacteria persisted, indicating that inadequate penetration alone cannot explain treatment failure. Together, these complementary datasets provide strong support for a model in which antibiotic tolerance is a key determinant of clinical outcome in KLAs.

We agree with the authors that the abscess microenvironment likely plays a critical role in modulating bacterial physiology and therapeutic response. In our study, spatial analyses revealed heterogeneity within infected tissue, including necrotic regions enriched for host-derived factors, which may contribute to the induction or maintenance of tolerance. The authors’ suggestion that percutaneous drainage may improve outcomes by altering this microenvironment is therefore well taken. In addition to reducing bacterial burden, drainage may influence local conditions such as oxygenation, nutrient availability, and inflammatory signaling, all of which are known to impact antibiotic susceptibility (3–10). Determining how these host-driven factors regulate tolerance will be an important area for future investigation.

We also appreciate the authors’ discussion of the structural complexity of human KLAs, including fibrotic encapsulation and septation, which may not be fully recapitulated in murine models. While our model captures key features of disseminated infection and abscess formation, we agree that additional models, including those that better reflect the architecture of human disease, will be valuable. Integrating such systems with quantitative and spatial approaches to measure antibiotic exposure and bacterial physiology will help further define the mechanisms underlying treatment failure.

In summary, the clinical observations reported by Gong et al. provide important validation of our findings and underscore the translational relevance of antibiotic tolerance in KLAs. These studies collectively highlight the need to move beyond antibiotic susceptibility alone and to better understand how the host environment shapes bacterial responses to therapy.

## Data Availability

There are no data underlying this work.

## References

[1] Z. Gong , Antibiotic tolerance coupled with limited benefit from drainage: Clinical challenges in Klebsiella pneumoniae liver abscess. Proc. Natl. Acad. Sci. U.S.A. **123**, e2606316123 (2026).42546193 10.1073/pnas.2606316123

[2] M. Angeles-Solano , Antibiotics accumulate in Klebsiella pneumoniae liver abscesses but fail to eliminate antibiotic-tolerant populations. Proc. Natl. Acad. Sci. U.S.A. **122**, e2524436122 (2025). 41296732 10.1073/pnas.2524436122 PMC12685145

[3] G. Borriello , Oxygen limitation contributes to antibiotic tolerance of *Pseudomonas aeruginosa* in biofilms. Antimicrob. Agents Chemother. **48**, 2659–2664 (2004). 15215123 10.1128/AAC.48.7.2659-2664.2004 PMC434183

[4] S. E. Rowe , Reactive oxygen species induce antibiotic tolerance during systemic *Staphylococcus aureus* infection. Nat. Microbiol. **5**, 282–290 (2020). 31819212 10.1038/s41564-019-0627-y PMC6992501

[5] S. Ronneau, C. Michaux, S. Helaine, Decline in nitrosative stress drives antibiotic persister regrowth during infection. Cell Host Microbe **31**, 993–1006.e6 (2023). 37236190 10.1016/j.chom.2023.05.002

[6] Y. Liu , Immune activation of the host cell induces drug tolerance in *Mycobacterium tuberculosis* both in vitro and in vivo. J. Exp. Med. **213**, 809–825 (2016). 27114608 10.1084/jem.20151248 PMC4854729

[7] J. R. Raneses, A. L. Ellison, B. Liu, K. M. Davis, Subpopulations of stressed yersinia pseudotuberculosis preferentially survive doxycycline treatment within host tissues. mBio **11**, e00901-20 (2020). 32753491 10.1128/mBio.00901-20 PMC7407081

[8] F. Peyrusson , Intracellular *Staphylococcus aureus* persisters upon antibiotic exposure. Nat. Commun. **11**, 2200 (2020). 32366839 10.1038/s41467-020-15966-7 PMC7198484

[9] S. Helaine , Internalization of Salmonella by macrophages induces formation of nonreplicating persisters. Science **343**, 204–208 (2014). 24408438 10.1126/science.1244705 PMC6485627

[10] S. Helaine, B. P. Conlon, K. M. Davis, D. G. Russell, Host stress drives tolerance and persistence: The bane of anti-microbial therapeutics. Cell Host Microbe **32**, 852–862 (2024). 38870901 10.1016/j.chom.2024.04.019 PMC11446042

